# Conversion from Alloplastic to Autologous Breast Reconstruction: What Are the Inciting Factors?

**DOI:** 10.1177/22925503221107214

**Published:** 2022-06-15

**Authors:** Brendon Bitoiu, Sofie Schlagintweit, Zach Zhang, Esta Bovill, Kathryn Isaac, Sheina Macadam

**Affiliations:** 8166 University of British Columbia, Vancouver, BC, Canada

**Keywords:** alloplastic breast reconstruction, autologous breast reconstruction, breast reconstruction, breast salvage

## Abstract

**Introduction:** Failure of alloplastic breast reconstruction is an uncommon occurrence that may result in abandonment of reconstructive efforts or salvage with conversion to autologous reconstruction. The purpose of this study was to identify factors that predict failure of alloplastic breast reconstruction and conversion to autologous reconstruction. **Methods:** A retrospective chart review was conducted of patients who underwent mastectomy and immediate alloplastic breast reconstruction between 2008 and 2019. Inclusion criteria included patients 18 years or older who underwent initial alloplastic reconstruction with a minimum of 3-year follow-up. Data collected included age, body mass index, cancer type, surgical characteristics, neo/adjuvant treatment details, and complications. Results were analyzed using Fischer's exact test, *t*-test, and multivariate logistic regression. **Results:** A total of 234 patients met inclusion criteria. Of those, 23 (9.8%) required conversion from alloplastic to autologous reconstruction. Converted patients had a mean age of 50.1 ± 8.5. The time from initial alloplastic reconstruction to conversion was 30.7 months. The most common reasons for conversion included soft tissue deficiency (48%), infection (30%), and capsular contracture (22%). Patients were converted to deep inferior epigastric perforator flap (DIEP; 52%), latissimus dorsi flap with implant (26%), and DIEP with implant (22%). Multivariate logistic regression modeling identified radiation (OR 8.4 [CI = 1.7-40.1]) and periprosthetic infection (OR 14.6 [CI = 3.4-63.8]) as predictors for conversion. **Conclusions:** Among patients undergoing mastectomy with immediate alloplastic breast reconstruction, those treated with radiation have 8.4 greater odds of conversion and those with a periprosthetic infection have 14.6 greater odds for conversion to an autologous reconstruction.

## Résumé

**Introduction :** L’échec d’une reconstruction mammaire alloplastique est un événement peu fréquent qui peut aboutir à un abandon des efforts de reconstruction ou à une solution de secours avec une conversion en reconstruction autologue. L’objectif de cette étude était d’identifier les facteurs prédisant l’échec de la reconstruction mammaire alloplastique et sa conversion en reconstruction autologue. **Méthodes :** Une étude rétrospective a été menée sur les dossiers de patientes ayant subi une mastectomie suivie d’une reconstruction mammaire alloplastique immédiate entre 2008 et 2019. Les critères d’inclusion étaient notamment des patientes âgées d’au moins 18 ans ayant subi une reconstruction alloplastique initiale avec un suivi minimum de 3 ans. La collecte de données a inclus : l’âge, l’indice de masse corporelle (IMC), le type de cancer, les caractéristiques chirurgicales, les détails sur le traitement adjuvant/néoadjuvant et les complications. Les résultats ont été analysés à l’aide du test *t* exact de Fischer, un test *t* et une analyse de régression logistique multifactorielle. **Résultats :** Un total de 234 patientes a satisfait les critères d’inclusion. Parmi ces patientes, 23 (9,8%) ont nécessité la conversion de la reconstruction alloplastique en reconstruction autologue. Les patientes « converties » avaient un âge moyen de 50,1 ± 8,5 ans. Le délai séparant la reconstruction alloplastique initiale et la conversion était de 30,7 mois. Les motifs les plus fréquents de conversion étaient une déficience des tissus mous (48%), une infection (30%) et la formation d’une coque (22%). Les techniques de conversion utilisées étaient la technique dite DIEP ou AEIP (artère épigastrique inférieure profonde, 52%), un lambeau du grand dorsal avec implant (26%), et la technique DIEP avec implant (22%). Une modélisation d’analyse de régression logistique multifactorielle a identifié les facteurs prédictifs de conversion suivants : irradiation (rapport de cotes = 8,4 [IC = 1,7-40,1]) et l’infection périprothétique (RC = 14,6 [IC = 3,4 à 63,8]). **Conclusions :** Parmi les patientes subissant une mastectomie avec reconstruction mammaire alloplastique immédiate, celles qui ont reçu une radiothérapie avaient un risque de conversion 8,4 fois plus grand et les patientes ayant une infection périprothétique avaient un risque 14,6 fois plus grand de conversion en reconstruction autologue.

## Introduction

Alloplastic breast reconstruction is the most common method of postmastectomy reconstruction.^1^ Despite improvements in prosthesis design and techniques, complications are reported as high as 52%.^2–6^ These include capsular contracture, seroma, implant rupture, infection, and poor longevity with a need for additional revision surgeries.^6,7^

Perioperative or long-term complications may result in conversion from alloplastic to autologous reconstruction or abandonment of breast reconstructive efforts. Risk factors for failure of alloplastic reconstruction include larger breast size, active smoking, implant exposure, radiation, and periprosthetic infection.^8–14^ Reconstruction failure attributed to patient-driven motivation from dissatisfaction with implant-based reconstruction or from physical discomfort is also described.^9,13,15–17^

Conversion to an autologous reconstruction has been shown to improve the aesthetic result, patient satisfaction, and quality of life in these difficult cases.^9,13,14^ The purpose of this study was to determine the incidence of alloplastic failure with salvage by autologous reconstruction and predictors of conversion from alloplastic to autologous reconstruction in a single tertiary care center. These results will aid preoperative patient counseling, and guide patient selection for breast reconstruction.

## Methods

The study received approval from the University of British Columbia Institutional Research Ethics Board (H20-03666). A retrospective chart review was performed of patients who had undergone mastectomy and immediate alloplastic breast reconstruction between January 2008 and December 2019 with at least a 3-year follow-up from 1 university-affiliated center. Inclusion criteria included patients 18 years or older who underwent an initial alloplastic reconstruction under the care of the 2 senior breast reconstructive surgeons. All patients who underwent initial autologous breast reconstruction had missing data or had <3 years of follow-up were excluded from the cohort.

The primary outcomes were frequency of conversion from alloplastic to autologous breast reconstruction and factors predictive of failure of alloplastic breast reconstruction and subsequent conversion to autologous reconstruction.

Data was collated from patient medical records. Baseline patient characteristic data included age, body mass index (BMI; where we define high BMI as >30), smoking status, tumor diagnosis, genetics, and treatment requirements (chemotherapy and/or radiation). Perioperative data included procedure type, tissue expander/implant size, indication for the procedure, laterality, need for axillary lymph node dissection, and mastectomy weight. A complication profile of alloplastic reconstruction was recorded including malposition, capsular contracture, contour irregularities (defined as rippling or irregularity of shape due to difference in skin and soft tissue thickness), implant rippling, seroma, hematoma, mastectomy flap nerosis (MFN), implant rupture, removal of tissue expander/implant for exposure, and infection (defined by a clinical diagnosis with a need for antibiotics). Indications for, and type of, conversion were collected including malposition, capsular contracture, seroma, MFN, implant rupture, reexcision for positive margins, infection, soft tissue deficiency (defined as an inadequate/insufficient skin envelope to create the breast mound or inadequate thickness), and flap type.

## Statistical Analysis

Descriptive statistics were used to outline the clinical characteristics of patients in the 2 comparison groups, that is, successful alloplastic reconstruction was defined as the “nonconversion” group, and failed alloplastic reconstruction with conversion to autologous was defined as the “conversion” group. Continuous variables are presented as means with standard deviations and were compared using the Mann-Whitney *U*-test. For binary and categorical variables, Fischer's exact test was applied. A multivariate analysis was performed using a logistic regression model with forward conditional selection was used to determine predictive factors associated with conversion to autologous reconstruction. Risk factors for failed reconstruction and subsequent conversion were determined a priori based on the literature.^1,8,14^ Predictors considered in the multivariate regression model included radiation therapy, perioperative infection, and MFN. Results were presented as odds ratio with 95% confidence intervals. All tests were 2-tailed and statistical significance was defined as *p* *<* .05. The analysis confirmed 82% power with the study sample size. Data analyses were conducted with IBM SPSS Statistics Version 24 software (SPSS).

## Results

Over the 11-year study period, 243 patients (324 breasts) were included. Of these patients, 23 (9.8%) patients (40 breasts) required conversion from alloplastic to autologous reconstruction. The mean time from alloplastic reconstruction to conversion to autologous tissue was 30.7 months (range 4-134 months). Study population characteristics are outlined in Table 1. The total number of procedures was 2.1 ± 1.1 for the nonconversion group and 4.7 ± 2.5 for the conversion group. Initial operative details including type of mastectomy, staged versus nonstaged procedure, and the plane of reconstruction is outlined in Table 2.

**Table 1.** Comparison of perioperative factors between alloplastic breast reconstruction patients that require conversion to autologous reconstruction versus those that do not require conversion.

Variable	No conversion	Conversion	*p*-value
Age (years)	n = 211 50.1 ± 10.8	n = 23 50.1 ± 8.5	.86*
Body mass index (kg/m²)	n = 209 23.4 ± 3.91	n = 23 25.6 ± 5.4	.04*
Smoking Yes No	n = 211 5 (2.4%) 206 (97.6%)	n = 23 1 (4.3%) 22 (95.7%)	.47†
Initial procedure indication Therapeutic Prophylactic	n = 211 198 (93.8%) 13 (6.2%)	n = 23 22 (95.7%) 1 (4.3%)	.99†
Laterality Unilateral surgery Bilateral surgery	n = 211 98 (46.4%) 113 (53.6%)	n = 23 7 (30.4%) 16 (69.6%)	.19†
Staged Yes No			
Axillary lymph node dissection Yes No	n = 209 18 (8.6%) 191 (91.4%)	n = 16 4 (25.0%) 12 (75.0%)	.06†
Mastectomy weight (g)	n = 202 459.9 ± 263.7	n = 19 534.2 ± 263.9	.18*
Tumor diagnosis In situ Invasive None	n = 209 38 (18.2%) 162 (77.5%) 9 (4.3%)	n = 22 8 (36.4%) 14 (63.6%) 0 (0%)	.14‡
Chemotherapy Yes No	n = 194 79 (40.7%) 115 (59.2%)	n = 23 13 (56.5%) 10 (43.5%)	.18†
Radiation Yes No	n = 195 91 (46.7%) 104 (53.3%)	n = 23 17 (73.9%) 6 (26.1%)	**.02†**
Infection Yes No	n = 211 6 (2.8%) 205 (97.2%)	n = 23 9 (39.1%) 14 (60.9%)	**<.01†**
Mastectomy flap necrosis Yes No	n = 211 21 (10.0%) 190 (90.0%)	n = 23 15 (65.2%) 8 (34.8%)	**<.01†**
Total number of procedures	n = 211 2.1 ± 1.1	n = 23 4.7 ± 2.5	<.01*

Bold values specify statistical significance. *Mann-Whitney *U*-test.

†Fisher's exact *t*-test.

‡ Freeman-Halton extension of Fisher's exact *t*-test.

**Table 2.** Initial operative details of patients who required conversion of alloplastic to autologous breast reconstruction.

Variable	Conversion
Staged operation Yes (TE) No (DTI)	n = 23 20 (86.9%) 3 (13.1%)
Plane Subpectoral Prepectoral	n = 23 23 (100%) 0 (0%)
Type of mastectomy SSM NSM Non-SSM	n = 23 15 (65.2%) 4 (17.4%) 4 (17.4%)

Abbreviations: TE, echo time; DTI, diffusion tensor imaging; SSM, skin-sparing mastectomy; NSM, nipple-sparing mastectomy.

A comparison of perioperative risk factors for conversion to autologous tissue is outlined in Table 3. Univariate analysis identified the following risk factors for conversion: high BMI (*p* = .04), radiation exposure (*p* = .02), perioperative infection (*p* < .01), and MFN (*p* < .01) (Table 1). Of the patients who underwent radiation therapy, 5 (n = 17, 29%) and 12 (n = 17, 71%), underwent neoadjuvant and adjuvant radiation therapy, respectively. Smoking status, initial procedure indication, need for axillary lymph node dissection, mastectomy weight, tumor diagnosis, and need for chemotherapy did not differ between groups.

**Table 3.** Multivariate binary logistic regression model of perioperative factors associated with the conversion of alloplastic to autologous breast reconstruction.

Variable	Odds ratio (95% confidence interval)	*p*-value
Variables in the model		
Radiation	8.4 (1.7-40.1)	**<.01**
Infection	14.6 (3.4-63.8)	**<.01**
Variables not in the model		
Body mass index		.38
Axillary lymph node dissection		.48
Mastectomy flap necrosis		.86

The most common complications reported in patients who underwent conversion to autologous tissue included contour irregularities (n = 11, 48%), MFN (n = 8, 39%), and infection (n = 8, 39%). The most common complications for patients who did not undergo conversion included contour irregularities (n = 25, 12%), malposition (n = 24, 11%), and MFN (n = 21, 10%) (Table 4).

**Table 4.** Complication profile of alloplastic breast reconstruction.

Complication	No conversion (n = 211)	Conversion (n = 23)	Overall (n = 234)
Malposition	24 (11.4%)	4 (17.4%)	28 (12.0%)
Capsular contracture	17 (8.1%)	6 (26.1%)	23 (10.0%)
Contour irregularities	25 (11.8%)	11 (47.8%)	36 (15.4%)
Implant rippling	6 (2.8%)	0 (0%)	6 (2.6%)
Seroma	5 (2.4%)	3 (13.0%)	8 (3.4%)
Hematoma	4 (1.9%)	1 (4.3%)	5 (2.1%)
MFN	21 (10.0%)	8 (39.1%)	29 (12.4%)
Implant rupture	6 (2.8%)	2 (8.7%)	8 (3.4%)
Removal of echo time (TE)/implant for exposure	1 (0.5%)	0 (0%)	1 (0.4%)
Infection	6 (2.8%)	9 (39.1%)	15 (6.4%)

In the multivariate analysis of perioperative factors, radiation exposure (OR 8.4 [1.7-40.1], *p* < .01) and periprosthetic infection (OR 14.6 [3.4-63.8], *p* < .01) were significantly associated with the conversion of alloplastic to autologous breast reconstruction. BMI, need for axillary lymph node dissection and presence of MFN were not significantly associated with conversion (Table 3).

Among the patients requiring conversion to autologous breast reconstruction, 52% were converted to deep inferior epigastric perforator flap (DIEP), 26% to latissimus dorsi flap with an implant, and 22% to DIEP with an implant (Table 5). Reasons for conversion to autologous reconstruction included soft tissue deficiency (n = 11, 48%), infection (n = 7, 30%), capsular contracture (n = 5, 22%), MFN (n = 3, 13%), malposition (n = 2, 9%), seroma (n = 2, 9%), implant rupture (n = 1, 4%), and reexcision for positive margins (n = 1, 4%) (Table 5).

**Table 5.** Conversion to autologous descriptive values.

Variable	
Time to conversion	30.7 mo (range: 4-134 mo)
Procedure type	n (%)
- DIEP	12 (52.2%)
- DIEP + implant	5 (21.7%)
- Latissimus dorsi flap + implant	6 (26.1%)
Indication for conversion	n (%)
- Malposition	2 (8.7%)
- Capsular contracture	5 (21.7%)
- Seroma	2 (8.7%)
- MFN	3 (13.0%)
- Implant rupture	1 (4.3%)
- Reexcision for positive margins	1 (4.3%
- Infection	7 (30.4%)
- Soft tissue deficiency	11 (47.8%)

Abbreviations: DIEP, deep inferior epigastric perforator flap;

## Discussion

In this single-center 11-year retrospective review of patients treated with mastectomy and immediate alloplastic reconstruction, the rate of conversion from alloplastic to autologous reconstruction was 9.8%. Perioperative factors that were significantly associated with conversion of alloplastic to autologous breast reconstruction included radiation exposure and infection. The most common autologous salvage procedures were DIEP flap, latissimus dorsi flap with an implant, and DIEP flap with an implant.

Radiation exposure being predictive of conversion is congruent with the previous literature supporting its association with increased complication rates and high rates of reconstructive failure in implant-based reconstruction.^18–21^ Chest wall irradiation may induce both acute and chronic changes with the latter manifesting as varying degrees of fibrosis, soft tissue deficiency, induration, permanent skin retraction, and decreased wound healing.^18^ Increased risks of capsular contracture, infection, expander extrusion, and wound healing complications associated with radiation, which range in incidence from 5% to 48%,^22^ are important considerations for reconstructive planning and shared decision-making.^8,18^

Radiation may be performed during the tissue expansion phase or after exchange for the permanent implant. Lower failure rates have been reported with radiation after exchange for the permanent implant^18,21,23,24^ and failure may be as high as 40% when radiation is performed during the tissue expansion phase.^24,25^ This may be a result of skin thinning and resultant expander extrusion. The second stage procedure involving radiated breast tissue may further increase wound healing complications leading to reconstructive failure.^18^ Capsular contracture rates also differ when considering the temporal nature of the radiation treatment.^9^ Cordeiro et al^21^ reported Baker Grade-III/IV capsular contracture in 17% of patients radiated during the tissue expansion phase compared to 51% of patients radiated after placement of the permanent implant. In our cohort, 17 patients underwent radiation therapy, of which 76% received radiation during the tissue expansion phase, 24% received radiation prior to mastectomy, and none received radiation to a permanent implant. This highlights both acute perioperative and long-standing radiation-induced changes that can lead to failure at various stages of the reconstruction.

Periprosthetic infection was also identified as an important predictor for conversion. Literature rates for infection after implant-based reconstruction have been reported from 1% to 35%.^26,27^ This large variability may exist because there is no consensus on a universal definition for perioperative infection.^28^ Historically, management of cellulitis, periprosthetic infection, or exposure required implant removal.^6^ However, currently traditional management may include attempts at salvage prior to removal of the device. This type of management may include systemic antibiotics, aspiration of periprosthetic fluid, and/or operative intervention with the placement of a new device.^29^ The majority of these infections occur from 31 days to 1-year postoperatively with radiotherapy and elevated BMI being identified as independent risk factors for late surgical site infections.^30^ In our series, we found that 60% of all periprosthetic infections were in the converted group. Of this group (n = 9), 2 cleared the infection with device exchange and intravenous antibiotics, and 7 eventually led to reconstructive failure. Of these 7 patients, 6 patients were managed with intravenous antibiotics and operative washout, whereas 1 patient was managed with intravenous antibiotics alone. In addition to radiation, the infection may also be associated with subsequent capsular contracture, which was reported in 26% of our conversion patients.^31^ This aligns with the literature also suggesting a correlation between bacterial presence on the implant and later capsular contracture.^6,32^

Numerous additional perioperative risk factors have been associated with adverse breast reconstruction outcomes including smoking status, BMI, diabetes mellitus, age, chemotherapy, and breast size. There is conflicting evidence on the impact of chemotherapy timing on the incidence of complications.^33–35^ Chemotherapy was not found to be associated with conversion in our series. Smoking is considered a universal risk factor for any surgery-associated complication, including breast reconstruction. Smokers are found to have higher rates of incidence of mastectomy flap necrosis, rates of infection, and reconstructive failure than nonsmokers.^11^ In this study, there was a very small cohort of active smokers (2.5%). We suspect this as the likely reason for smoking not being a predictive factor for breast reconstruction failure and subsequent conversion. BMI was not found to be associated with conversion once adjusted for other important variables.

Given the retrospective cohort study designs, there are several limitations, including the potential for unaccounted confounding factors. Evaluation of patient comorbidities, including diabetes mellitus and a more robust smoking assessment, may have allowed for a more detailed review of these risk factors in our analysis. Moreover, our analysis did not record patients who failed alloplastic reconstruction and did not proceed with autologous reconstruction. This report is also limited by its single-center study design and thus may lack external validity and generalizability.

## Conclusion

Among patients undergoing mastectomy with immediate breast reconstruction, those treated with radiation have 8.4 greater odds of conversion and those with a periprosthetic infection have 14.6 greater odds for conversion to an autologous method of reconstruction. The overall conversion rate in a single center with a 3-year follow-up is approximately 10%. These data will help surgeons counsel future patients about their risks of requiring an autologous reconstruction after deciding to proceed with an alloplastic form of reconstruction initially.
